# Identification of New Antifungal Compounds Targeting Thioredoxin Reductase of *Paracoccidioides* Genus

**DOI:** 10.1371/journal.pone.0142926

**Published:** 2015-11-16

**Authors:** Ana Karina Rodrigues Abadio, Erika Seki Kioshima, Vincent Leroux, Natalia Florêncio Martins, Bernard Maigret, Maria Sueli Soares Felipe

**Affiliations:** 1 Department of Biology, University of Mato Grosso State - UNEMAT, Nova Xavantina, Mato Grosso, Brazil; 2 Department of Clinical Analysis and Biomedicine, State University of Maringá, Maringá, Paraná, Brazil; 3 LORIA, Lorraine University, Nancy, France; 4 Embrapa - Genetic Resources and Biotechnology, Brasília, Distrito Federal, Brazil; 5 Department of Cellular Biology, University of Brasília - UnB, Brasília, Distrito Federal, Brazil; Louisiana State University, UNITED STATES

## Abstract

The prevalence of invasive fungal infections worldwide has increased in the last decades. The development of specific drugs targeting pathogenic fungi without producing collateral damage to mammalian cells is a daunting pharmacological challenge. Indeed, many of the toxicities and drug interactions observed with contemporary antifungal therapies can be attributed to “nonselective” interactions with enzymes or cell membrane systems found in mammalian host cells. A computer-aided screening strategy against the TRR1 protein of *Paracoccidioides lutzii* is presented here. Initially, a bank of commercially available compounds from Life Chemicals provider was docked to model by virtual screening simulations. The small molecules that interact with the model were ranked and, among the best hits, twelve compounds out of 3,000 commercially-available candidates were selected. These molecules were synthesized for validation and *in vitro* antifungal activity assays for *Paracoccidioides lutzii* and *P*. *brasiliensis* were performed. From 12 molecules tested, 3 harbor inhibitory activity in antifungal assays against the two pathogenic fungi. Corroborating these findings, the molecules have inhibitory activity against the purified recombinant enzyme TRR1 in biochemical assays. Therefore, a rational combination of molecular modeling simulations and virtual screening of new drugs has provided a cost-effective solution to an early-stage medicinal challenge. These results provide a promising technique to the development of new and innovative drugs.

## Introduction

The incidence and prevalence of invasive fungal infections (IFI) have been important causes of morbidity and mortality, especially in the large population of immunocompromised patients [1], [2]. The IFI incidence has also increased significantly and represents a serious public health problem, because it is associated with increased morbidity and prolonged length of hospital stay and, consequently, high costs for critically ill patients [3], [4], [5]. The most important IFI agents involved in opportunistic mycoses are *Candida* spp., *Cryptococcus neoformans* and *Aspergillus* spp. [6], [7], [8], [9], whereas the most commonly endemic mycoses are due to *Histoplasma capsulatum*, *Paracoccidioides brasiliensis* and *Coccidioides* spp. [10], [11].

Paracoccidioidomycosis (PCM) is the systemic granulomatous mycosis caused by the fungus *P*. *brasilisiensis* [12]. Recent phylogenetic studies have established a new species within *Paracoccidioides* genus, named *Paracoccidioides lutzii* [13], [14]. The disease is restricted to Latin America, where ten million people are supposed to be infected with this fungus of which up to 2% may develop the disease [15], [16], [17]. The chronic form is the more frequent one among adult males and progresses slowly. The acute form is fast (weeks or months) and more severe, leading to high mortality levels [18]. Additionally, many patients present relapses, complications, and sequelae such as pulmonary fibrosis.

The PCM treatment is lengthy, commonly lasting more than six months. Despite many advances in antifungal drugs development during the past decade, the therapeutic arsenal to the PCM treatment is restricted to itraconazole, sulfamethoxazole-trimethoprim, and amphotericin B deoxycholate [18]. The more recent drugs, such as third generation azoles (voriconazole and posaconazole) [19], [20], echinocandins [21] and the lipid-associated formulations of amphotericin B [22], no showed great benefits as compared to older options. The randomized clinical trials performed with 35 patients concluded that treatment with voriconazole and itraconazole are equally effective [23]. *In vitro* experiments showed micafungin has no activity for the parasitic phase of *Paracoccidioides brasiliensis* [24] and there is no clinical reports of the echinocandins use to PCM treatment. Clinical experience with the lipid formulations of amphotericin B is scarce, and has been shown to be as effective as deoxycholate amphotericin B [25].

The search for drugs more specific, to tackle the worldwide resistance and to minimize the serious side effects problem, remains a major challenge [26], [27], [28], [29]. Besides classical routes for drug development, the pharmaceutical industry also has adopted traditionally technologies as high-throughput screening (HTS), especially from the biodiversity, for identification of novel lead compounds for a given molecular target, however, this strategy has been associated with high cost, long-time and low hit rate [30], [31], [32].

Post-genomic data of human pathogenic fungi, in combination with homology modeling, molecular dynamics and virtual screening/docking of small molecules, may improve the discovery process and allow the identification of new potential drug targets [33], [34], [28]. Docking computations are now acknowledged as efficient techniques in complement or in replacement of HTS [35], [36]. In this context, comparative genomics recently allowed the identification of ten potential drugs targets for eight human fungal pathogens [28]. One of those is the thioredoxin reductase (TRR1), a flavoprotein enzyme participating in the oxidative stress resistance, also involved in regulating DNA synthesis, methionine biosynthesis, cell growth, gene transcription and apoptosis [37], [38]. The *trr1* gene is essential in many pathogens and represents a broad-spectrum target for drug development [39]. This gene was experimentally demonstrated as essential in *C*. *neoformans* [39], [40], *Plasmodium falciparum* [41], *Staphylococcus aureus* [42], *Aspergillus fumigatus* [43] and *Candida albicans* [44]. In other hand, for the phytopathogenic fungus *Magnaporthe oryzae*, the *trr1* gene is no essential [45]. However, this is not a rule for all pathogens. Nevertheless, its continues to be a promising target for the disease treatment, including rice blast disease. Strains with mutation in *trr1* gene resulted in several attenuation in their ability to grow in rice cells and failed to produce spreading necrotic lesions on the leaf surface. Even not being essential in all organisms, the *trr1* gene performs important functions that allow the pathogen survival into the host.

The thioredoxin (Trx) and NADPH make up the thioredoxin system ubiquitously present in organisms from prokaryotes to mammals. Two types of TRRs have been characterized; one the low-molecular-mass isoform (~35 kDa), which is present in prokaryotes, plants, some parasites and fungi [46], [47], [48]. Another isoform, the high molecular mass (~55 kDa), is found in higher eukaryotes including human and parasites [49], [50]. TRRs have similar functions but mechanistically they are very different [51]. In addition, the isoforms display distinct protein structures, mainly in the catalytic site [52], [53]. The C-terminal redox center of *Plasmodium falciparum* and human thioredoxin reductase enzymes showed differences, which were sufficient to obtain specific inhibitors for parasite protein [54]; therefore, TRR1 is a selective drug target. Those data reinforce TRR1 as a promising target for structural based drug design [55], [54] against infectious disease.

This work describes the identification of specific TRR1 inhibitors providing antifungal activity against *Paracoccidiodes* spp. A diverse dataset of 3,000 small molecules from a commercial supplier were docked to the previously-published TRR1 3D model [28]. Twelve compounds were selected from the docking results and subsequently tested experimentally. Among the compounds tested, three showed inhibitory activity against the fungal enzyme and were patented [56], [57], [58]. One demonstrated both selectivity and high antifungal activity against *Paracoccidioides* species.

## Materials and Methods

### Protein Model Preparation

In the absence of solved experimentally structure, a preliminary 3D model of TRR1 structure of *P*. *lutzii* was generated by computer-aided molecular modeling [28]. According to the BLAST search performed on the entire PDB database, the TRR1 of *P*. *lutzii* showed good sequence identity with two templates, specifically 3ITJ (PDB ID) of *S*. *cerevisiae* (65% sequence identity) and 1VDC (PDB ID) of *Arabidopsis thaliana* (57% sequence identity). This model was further submitted to molecular dynamics simulations in order to gain a better relaxation and a more correct arrangement. For this purpose, the protein was first solvated with a (80 Å)^3^ box of TIP3P explicit water molecules [59]. Next, Cl^-^ ions were added for ensuring the electrostatic neutrality. The NAMD program version 2.6 was employed in conjunction with the CHARMM22 force field [60], [61] in order to simulate the ensemble of 47,543 atoms-system. The initial state for dynamics was generated from the model after 6,400 steps of conjugate gradient minimization followed by an equilibration stage of 500 ps. The simulations were carried out in the isobaric-isothermal ensemble, maintaining the pressure and the temperature at 1 atm and 300K, respectively, by using Langevin dynamics (damping parameter of 1 ps^-1^) and the Langevin piston approaches. The equations of motion were integrated with a 1 fs time step, using the r-RESPA algorithm [62] electrostatic forces at a slower 2 fs frequency. Long-range interactions were treated using the particle-mesh Ewald approach [63], with an 11 Å cut-off (switching distance 9 Å) for the real space calculation. The calculation of forces and motion equations was repeated to generate a trajectory corresponding to a simulation time of 100 ns. A conformation was recorded every 1 ps, generating a trajectory of 10,000 conformations. Conservation of the secondary structure elements along the molecular dynamic trajectories was checked by using the Timeline plug-in in VMD [64]. Pockets were detected using METAPOCKET [65], an algorithm that bases itself on several reliable pocket detection tools to combine their results and improve detection. Changes in pocket volume and surface (pocket descriptors) were monitored with MDPocket [66], a software capable of analyzing cavities topological changes during MD simulations.

### Ligand Data Preparation

The ligands used were from the *Life Chemicals* provider (http://www.lifechemicals.com). In order to focus virtual screening only on drug-like compounds, a filtering procedure was carried out with the FILTER software (http://www.eyesopen.com/filter) (Openeye Scientific Software, Inc.). Only compounds with a molecular weight between 350–650 Da and with a maximum of 25 rotamers were retained. Toxic and totally insoluble compounds (according to FILTER criteria) and those with more than two Lipinski rule-of-five [67] violations were also filtered out. Additionally, a chemical diversity property was applied with Tanimoto Index (T) calculation (Jaccard coefficient) [68], allowing the similarity measuring between two molecules by a chemical point. Molecules with T> 0.2 were eliminated. A total of 3,000 compounds were therefore selected and pre-processed into VSM-G [69] in order to automatically perform all ligand modeling steps prior required to docking. The 3D structures for all molecules were obtained from the CORINA software [70]. The molecules protonation states and atom names were corrected when needed for pH 7 and compatibility with the GOLD program respectively, using a script homemade.

### Virtual Screening Process

Docking simulations were performed using the GOLD software [71], which does semi-flexible docking using a genetic algorithm. Each candidate molecule was subjected to a maximum of 50 docking runs, with docking stopped in advance if the top 5 conformations converge within a 1.5 Å RMSD range. The cavity coordinates were defined using the center of mass defined by the Ligsite program [72]. The docking results were collected from each GOLD screening and used to build our working database consisting in the top 100 ranked compounds.

### Docking Analysis/Visualization

The visualization of the protein and ligands interactions was performed by LIGPLOT software [73].

### Expression of Recombinant Enzymes TRR1 and TRX1 from *P*. *lutzii*

The recombinant proteins TRR1 and TRX1 of *P*. *lutzii* (Pb01) were expressed in *E*. *coli* strain BL21 (λDE3) (Novagen) using pET21a synthetic plasmid (Epoch Life Science, inc.). Transformed cells were grown at 37°C in LB medium on reaching OD_600nm_ = 0.6 and incubated for 6 h at 37°C with 0.5 mM IPTG. The cell pellet was resuspended in lysis buffer (20 mM sodium phosphate pH 8.0, 300 mM sodium chloride and 20 mM imidazole). The cell suspension was sonicated and centrifuged at 8000xg, 20 min. The resulting supernatant was applied to a nickel-affinity column (Hi-Trap from GE Healthcare) as per the manufacturer’s instructions. TRR1 was purified by affinity chromatography and visualized by SDS-PAGE and western-blot.

### Bioassay

#### Antifungal agents

The following compounds were used for susceptibility tests: Fluconazole (Sigma-Aldrich), Amphotericin B (Sigma-Aldrich), F0876-0030, F1109-0100, F1806-0122, F3010-0057, F3222-4930, F3307-0033, F3307-0100, F3394-0412, F3394-0585, F3398-5211, F5652-2782 and F5754-0452 (Life Chemicals INC., Burlington, ON, Canada). Stock solutions of each drug were prepared utilizing RPMI 1640 (Sigma) buffered to pH 7.0 with 0.165 M morpholinepropanesulfonic acid (MOPS) buffer (Sigma) as the solvent. Dimethyl sulfoxide (DMSO) and Pluronic F127 (Sigma) were used for solubilization of Life Chemicals molecules. To establish that exposure to diluent (DMSO and Pluronic) did not affect the growth of the tested microorganisms fungi species were grown in the presence of diluent and compared with positive control (only RPMI 1640).

#### Organisms

The *in vitro* susceptibility tests were performed for seven pathogenic yeasts: two isolates from *P*. *lutzii* (Pb01 and 8334), three isolates from *P*. *brasiliensis* (Pb18, 1Mg14 and 1Mg15), *C*. *albicans* (ATCC 90028), *C*. *parapsilosis* (ATCC 22019), *C*. *glabrata* (ATCC 90030), *C*. *tropicalis* (ATCC 750) and *C*. *neoformans* (H99). Prior to testing, each strain of *Candida* spp., was subcultured in Sabouraud dextrose agar (SDA; Becton, Dickinson and Company, Sparks, MD) and incubated at 30°C for 1 day. *C*. *neoformans* was subcultured in Yeast Extract Peptone Dextrose Agar (YPD; Becton, Dickinson and Company, Sparks, MD) at 30°C for 2 days. Each isolate of *Paracoccidioides* spp. was subcultured in Fava-Netto medium [74] at 36°C and used on the 5^th^ day of culture.

#### Antifungal susceptibility assays

The MICs of the 12 molecules selected after virtual screening and the control antifungal agents were determined consistently using the broth microdilution methods developed by the Clinical and Laboratory Standards Institute.

M27-A3 (CLSI) [75]. The final concentrations of the antifungal agents studied ranged from 0.03 to 256 μg/mL for Life Chemical compounds and 0.03 to 4.0 μg/mL for fluconazole and amphotericin B. Briefly, the *Candida* species were pre-cultured in Sabouraud Dextrose agar (Difco^®^, Sparks, MD, USA) at 35°C for 24 hours. Suspensions were prepared in sterile saline solution (0.85%) with yeast concentrations adjusted to 0.5 to 2.5 x 10^3^ CFU/mL. RPMI-1640 (Sigma-Aldrich^®^, St. Louis, MO, USA) culture media was used and was buffered with 3-morpholinopropanesulfonic acid (Sigma-Aldrich^®^, St. Louis, MO, USA) pH 7.0 and supplemented with 2% glucose. The MIC results for all agents were read following 24 h of incubation at 35°C. All tests were performed in triplicate. A standard strain of *C*. *parapsilosis* ATCC 22019 was used as a control in the tests.

The MIC protocol was adapted to *Paracoccidioides* spp. and *C*. *neoformans* conditions. To *Paracoccidioides* isolates the modifications include differences in inoculum preparation and incubation periods. Briefly, the yeast were pre-cultured in Fava-Netto medium [74] at 35°C for five days. Suspensions were prepared in sterile saline solution (0.85%) with yeast concentrations adjusted to rom 0.5 to 2.5 × 10^5^ CFU/mL. The MIC plates were incubated at 35°C for 10 days [76]. All tests were performed in triplicate. To *C*. *neoformans* only the incubation period was modified to 48 hours of incubation at 35°C, as described in the document M27-A3 [75] to non-*Candida* yeasts.

Endpoint determination readings were performed visually. The results of minimum inhibitory concentrations (MICs) were determined as the lowest concentration of antifungal able to inhibit 50% of the cell growth, as compared to its respective positive control [75].

To Minimal Fungicide Concentration (MFC) determination, the aliquots from each well were withdrawn and Petri dish with SDA medium were inoculated for checking absence of fungal growth. These cultured microplates were incubated for 48 h at 35°C. Finally, MFC was defined as the lowest concentration of compounds that produced a reduction of CFU 99.9% compared to its respective positive control.

#### *In vitro* cytotoxicity assay

The compounds were tested for the cytotoxicity on murine macrophages cell line (J774) using modification of 3-(4,5-dimethyl-2-thiazolyl)-2,5- diphenyltetrazolium bromide (MTT) assay method [77]. The cells were seeded onto 96-well microtitre plates at the density of 2 × 10^4^ cells/well in Dulbecco’s modified Eagle’s medium (DMEM) supplemented with fetal bovine serum (FBS), at 10% (v/v) (fetal bovine serum, Gibco BRL, Invitrogen, Paisley, Scotland). The cells were cultured in a humidified atmosphere of 5% CO_2_ and 95% air at 37°C. After 24 h incubation, 200 μL of compounds (F0876-0030, F1806-0122 and F3307-0100) were added to row of the microtitre plates and several 2-fold serial dilutions, in order to give final concentrations ranging from 32 to 512μg/mL. The cells cultured with the compounds were incubated for 24 h in a humidified atmosphere of 5% CO_2_ and 95% air at 37°C. The freshly prepared MTT solution (5 mg/ml concentration in PBS) was added to each well and incubated at the condition as above for 2 h. The plates were centrifuged at 450xg for 5 min and the supernatant aspirated. The isopropanol was added into each well, allowed to stand for about 30 min to dissolve the formazan crystals. The plates were transferred to an ELISA microtitre plate reader at A_570nm_. The cytotoxicity was expressed as the percentage of cell viability compared to untreated control cells.

#### Thioredoxin reductase DTNB activity

The thioredoxin reductase 5,5′-dithiobis(2-nitrobenzoate) (DTNB) assay was used to measure thioredoxin reductase activity. The DTNB assay measures the ability of the TRR1 to be reduced by NADPH and in turn to reduce DTNB. The specific activities were determined using the molar extinction coefficient of TNB released during the reaction (13,600M^-1^ cm^-1^) [78], [79]. Briefly, the assay mixture contained 100 mM potassium phosphate, pH 7.0, 1 mM EDTA, 0.2 mM NADPH, 0.1 mg/mL bovine serum albumin and 0.25 mM DTNB. The DTNB-reduction was followed spectrophotometrically (Shimadzu) in a 1 mL assay at 25°C by monitoring the increase of A_412 nm_. TRR1 concentrations were fixed in all experiments at 0.09 μM. Purified recombinant thioredoxin/Trx1 enzyme (from *P*. *lutzii*) was used at concentrations ranged from 0.125 to 20.0 μM. The reaction was initiated by the addition of 0.2 mM NADPH. The apparent kinetic constants were determined by nonlinear regression of Michaelis-Menten plots using the GraphPad Prism 5.0 software (Inc., San Diego, CA, EUA). The measured activities were corrected by subtracting the velocities of the control reactions without thioredoxin. At each Trx1 concentration, three replicates were performed.

#### Inhibition of recombinant TRR1 enzyme by compounds

The compounds of Life Chemicals F0876-0030, F1806-0122 and F3307-0100 were tested as thioredoxin reductase inhibitors. Thioredoxin reductase activity assay was performed as described above. The substrate Trx1 was used at 2.0, 6.0 and 16.0 μM concentrations. In addition, the small molecules were added at concentrations of 32.5, 65 and 130 μM (F0876-0030); 155, 309 and 618 μM (F1806-0122); and 65, 130 and 260 μM (F3307-0100). Reactions were carried out in the presence and absence of the inhibitors. The kinetic parameters K_m_ and V_max_ were determined from the Michaelis-Menten equation.

## Results and Discussion

### Molecular Dynamics Simulations

**Fig 1:**
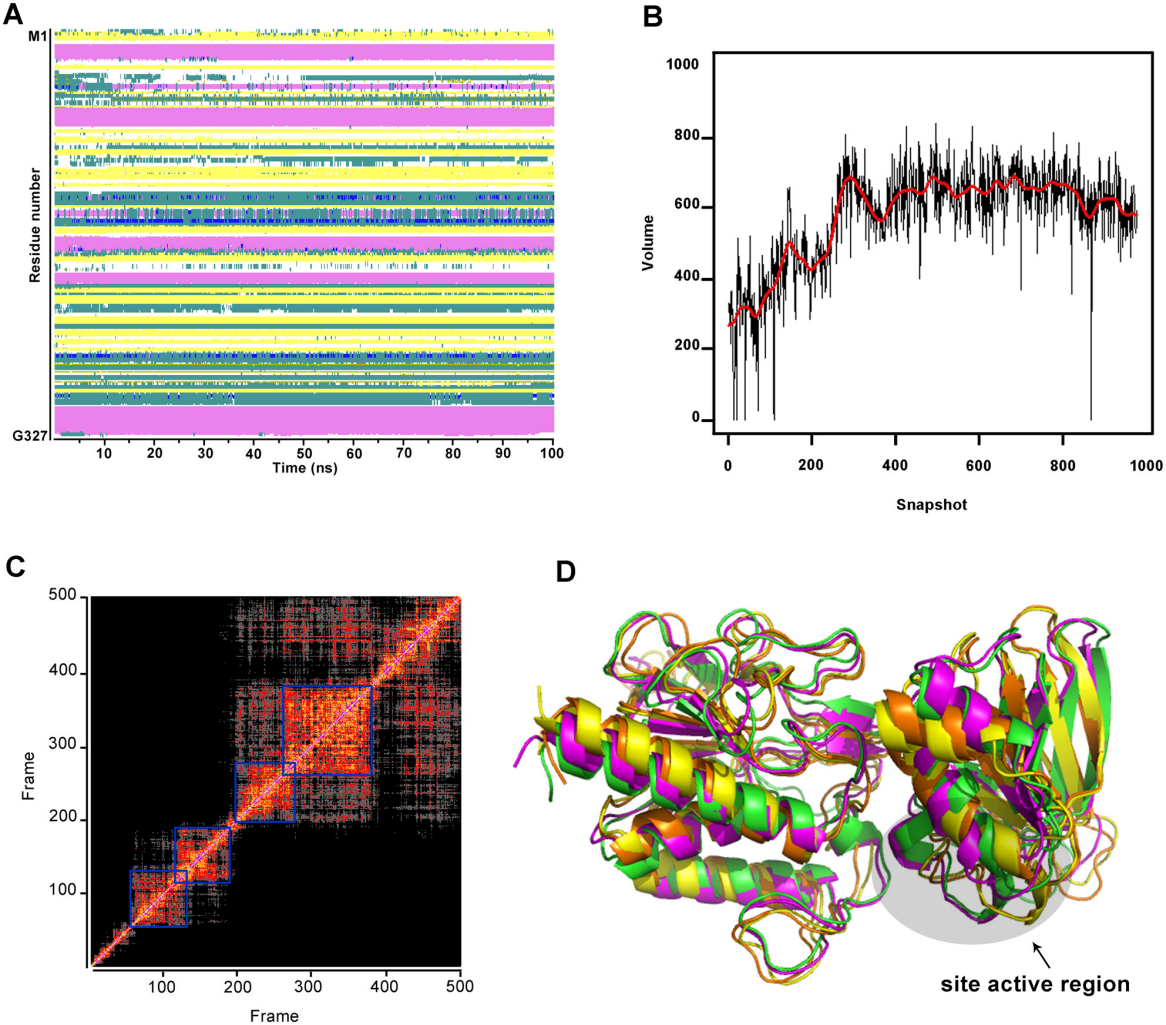
Molecular dynamic of *P*. *lutzii* TRR1 model. **(A)** Secondary structure elements evolution of the TRR1 model along MD trajectory; α-helices and β-sheets are shown in pink and yellow, respectively. **(B)** Evolution of the volume cavity of TRR1 model along MD trajectory. **(C)** RMSd (*Root Mean Square Deviation*) values plot of atoms from 500 conformations of TRR1 3-D model along MD trajectory. Each point on the graph represents the RMSd between the conformations, and the lowest RMSd values are close to the diagonal line. **(D)** 3D model of the four most probable conformations overlapped shown by colored lines.

The molecular model previously reported by Abadio et al. [28] was used for molecular dynamics and virtual screening of new inhibitory compounds. Prior to the virtual screening process, MD simulations were performed to (i) investigate the TRR1 model stability and (ii) to cluster TRR1 conformational spaces into a limited number of representative families. After simulations, the water box size was (77.5 Å)^3^ (compared to the initial (80 Å)^3^), showing that no significant change occurred in the system and in the water box stability. The folding stability of the model during the simulations was checked by monitoring secondary structure conservation over simulation time. It appears from Fig 1A that the overall protein shape is maintained, as soon it concerns the secondary structures which remain quite stable during the whole simulation time. The analysis shows that TRR1 model is stable enough to be next used for the virtual screening process.

TRR1 catalytic site contains the four amino acid residue sequence Cys-Ala-Thr-Cys, where these two highly conserved cysteine residues are present in the NADPH binding domain and are essential for its redox activity [80], [81], [51], [28]. Other residues present in the TRR1 active site are Ala151, Val152, Pro153 and Ile154 [28]. Plotting the evolution of the TRR1 binding site during the MD simulation shows (Fig 1B) that its geometrical characteristic (cavity volume) was stabilized after few ns of MD, with an average value around 600 Å^2^. As in many proteases the pocket volume presented a ‘breathing’ behavior, adopting a sinusoidal curve when plotted against time. This suggested that, even if the protein binding core remained stable during the simulation, the binding site accessibility seems to smoothly change and therefore may adapt to several kind of ligands. This result reinforces the overall analysis above concerning the TRR1 model and assuming that the molecule characteristics achieved enough stability during the MD simulation to provide good structural basis for the docking process.

One very useful way to expose relationships among the molecular configurations is to group or cluster them into subsets based on the similarity of their conformations. To classify the conformations coming from a MD trajectory into distinct groups clustering has been proved to be a useful procedure [82]. To visualize the clusters in our MD trajectory, a RMSd plot between all the frames was drawn. This plot shows that four clusters can be clearly identified. The 4 conformers considered as representative of these clusters are depicted (Fig 1C). The 3D model of the best four probable conformers were overlapped and showed by colored lines (Fig 1D). Their conformations are quite similar with RMSd values ranging between 2.4–4.0 Å. The domain-domain was conserved and most of the changes are observed in the unstructured regions and in the helix-sheet domain in which the helices positions are weakly different. The positions of the active site residues are almost conserved in the 4 conformers. Therefore, these 4 conformers, considered as exhibiting the main features of the protein flexibility, will be next used for performing an ensemble docking campaign [83], [84].

### Virtual Screening and Compounds Selection

**Fig 2:**
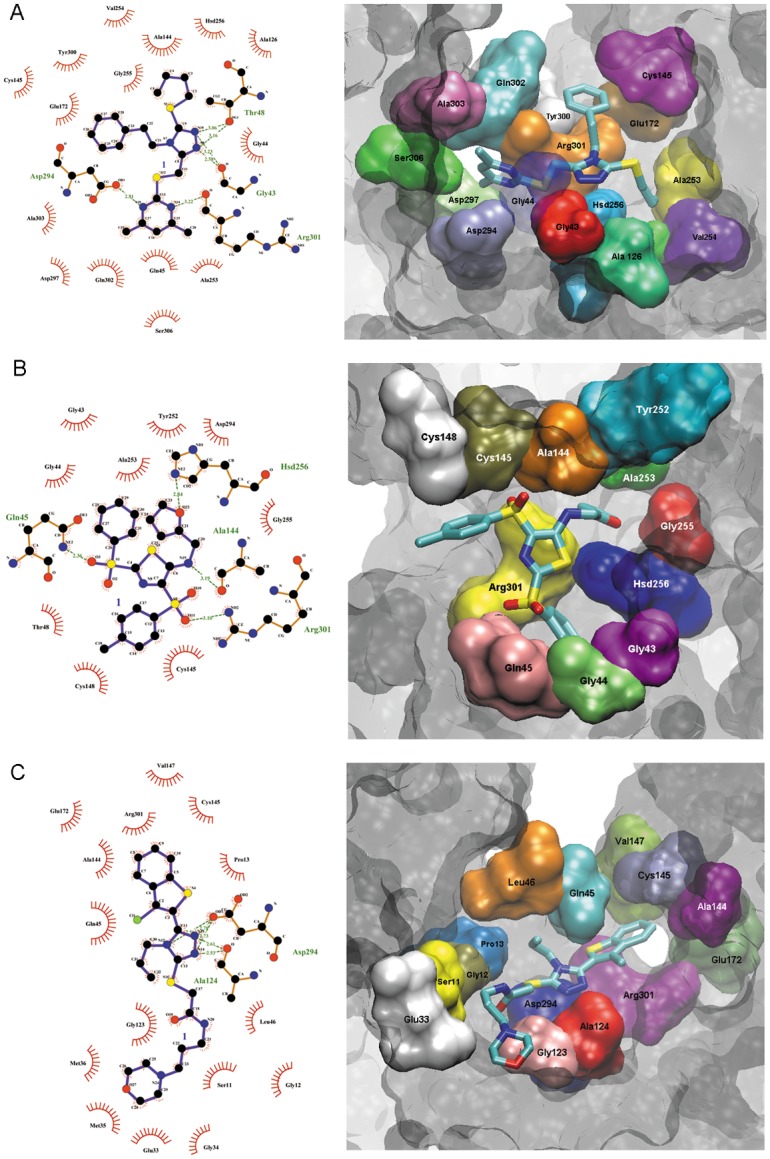
Docking interaction of *P*. *lutzii* TRR1 with ligands F0876-0030; F1806-0122 and F3307-0100. Schematic drawing of interactions between active site residues and the ligands using ligplot. The three compounds preferentially bind to Cys145 and/or Cys148 amino acids residues of the enzyme active site.

The selected 3,000 compounds from Life Chemicals were docked into the four representative protein conformations, as defined above. The ligands were ranked according to their docking scores. After analysis of the whole scores distribution, the best scored compounds (up to 70.00) were selected. From them, the top 12 compounds (Table 1) were retained to perform the antifungal tests experimentally. These organic compounds are structurally similar presenting, for example, molecular weight ranging from 413 to 589 Da. Most of them have in common carbonyl, sulfanyl and triazole groups. They have the capacity to perform interactions, as hydrogen bond and hydrophobic interaction, to form a complex with TRR1 target. Three of them, specifically F0876-0030, F1806-0122 and F3307-0100, perform hydrophobic interaction with relevant amino acids residues at the TRR1 active site (Fig 2).

**Table 1:** Top ranking ligands of synthetic compounds from Life Chemicals library after virtual screening against TRR1 using GOLD docking program software.

Life Chemicals	Ligand IUPAC	Gold Score (for TRR1 Conformation)			
Id	Name	1	2	3	4
F0876-0030	2-[4-Allyl-5-(3-chloro-benzo[b]thiophen-2-yl)-4H-[1,2,4]triazol-3-ylsulfanyl]-N-(3-morpholin-4-yl-propyl)-acetamide	91.55	78.13	74.28	72.62
F1806-0122	2-(5-Butylsulfanyl-4-phenethyl-4H-[1,2,4]triazol-3-ylmethylsulfanyl)-4,6-dimethyl-pyrimidine	79.28	75.00	81.30	77.38
F3307-0100	[2-Benzenesulfonyl-4-(toluene-4-sulfonyl)-thiazol-5-yl]-furan-2-ylmethyl-amine	89.74	76.42	79.86	72.84
F1109-0100	5-Cyano-4-(4-hydroxy-3-methoxy-phenyl)-2-methyl-6-(thiazol-2-ylcarbamoylmethylsulfanyl)-1,4-dihydro-pyridine-3-carboxylic acid allyl ester	86.41	74.60	76.32	74.81
F3010-0057	3-[5-(4-Methoxy-phenyl)-4H-[1,2,4]triazol-3-ylsulfanyl]-N-phenethyl-3-thiophen-2-yl-propionamide	90.37	74.23	75.22	78.30
F3222-4930	2-[2-(3,4-Dimethoxy-phenyl)-5-methyl-oxazol-4-ylmethylsulfanyl]-6-(4-methoxy-phenyl)-pyrimidin-4-ol	88.56	80.74	81.05	78.00
F3307-0033	5-Methylsulfanyl-4-(toluene-4-sulfonyl)-2-[2-(toluene-4-sulfonyl)-ethyl]-oxazole	81.21	76.36	79.72	74.60
F3394-0412	3-(3-Allyl-5-furan-2-yl-4-oxo-3,4-dihydro-thieno[2,3-d]pyrimidin-2-ylsulfanyl)-N-benzo[1,3]dioxol-5-ylmethyl-propionamide	87.92	78.64	81.73	82.42
F3394-0585	N-[5-(4-Chloro-phenyl)-[1,2,4]thiadiazol-3-yl]-2-(4-oxo-5-phenyl-4,5-dihydro-1H-pyrazolo[3,4-d]pyrimidin-6-ylsulfanyl)-acetamide	86.37	78.70	73.39	76.80
F3398-5211	5-Methyl-1-(5-methyl-2-p-tolyl-oxazol-4-ylmethyl)-1H-[1,2,3]triazole-4-carboxylic acid 4-methylsulfanyl-benzylamide	81.21	79.84	80.83	76.09
F5652-2782	2-(6-Oxo-3-p-tolyl-6H-pyridazin-1-yl)-N-[2-(3-phenyl-[1,2,4]triazolo[4,3-b]pyridazin-6-yloxy)-ethyl]-propionamide	87.09	79.33	73.37	87.24
F5754-0452	2-(4-Methyl-thiazol-2-yl)-3-oxo-4-[4-(2-phenoxymethyl-benzoimidazol-1-ylmethyl)-piperidin-1-yl]-butyronitrile oxalate	79.90	76.45	79.01	81.64

### Bioassay

**Fig 3:**
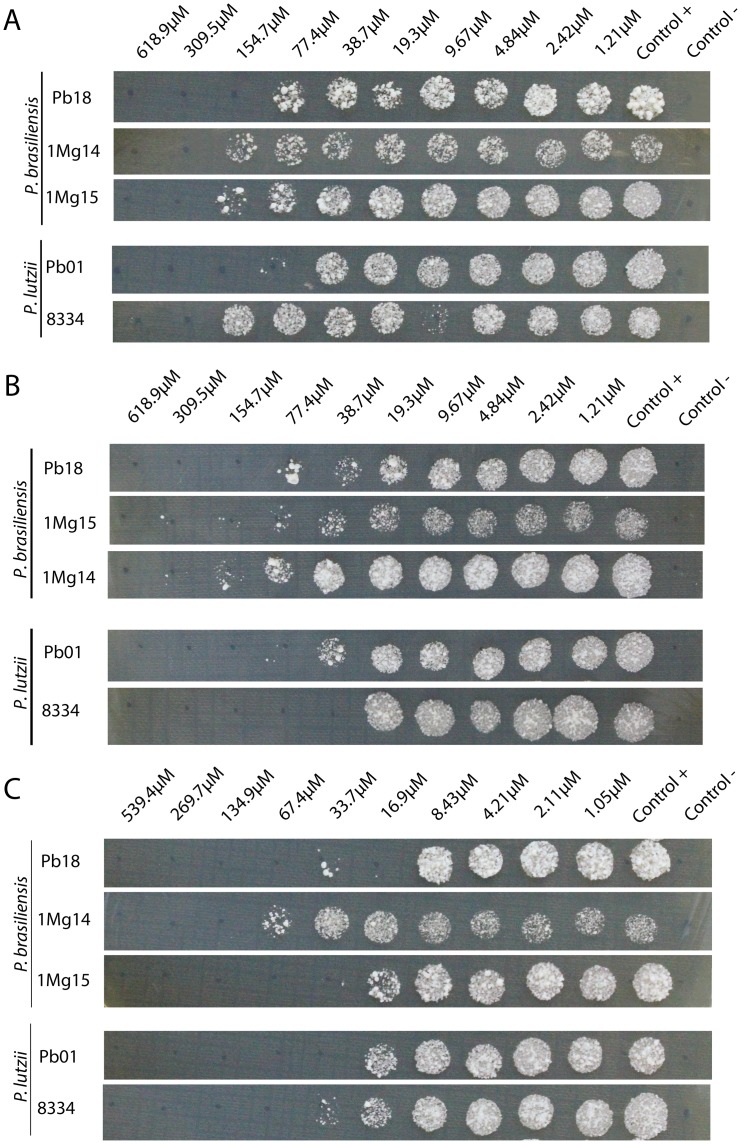
Minimum Fungicide Concentration of F0876-0030 (A), F1806-0122 (B) and F3307-0100 (C) against several *Paracoccidioides* isolates. The fungicide activity determination was performed by subculture of the MIC concentrations in brain heart infusion (BHI) agar plates, at 37C for 5 days. The three compounds presented fungicidal activity for all isolates, particularly the F3307-0100. The MCF values for this compound ranged between 16.9 μM to 134.9 μM for *P*. *brasiliensis* isolates and 33.7 μM to 67.4 μM against *P*. *lutzii* isolates.

From 12 compounds tested, 3 of them showed *in vitro* antifungal activity: F0876-0030, F1806-0122 and F3307-0100. The best antifungal activity performed by two compounds: F1806-0122 and F3307-0100 (Table 2). For these compounds, the MIC values against *P*. *brasiliensis* and *P*. *lutzii* were equal, 8 μg/mL and 16 μg/mL, respectively. However, the F3307-0100 compound showed relevant fungicide activity against several *Paracoccidioides* isolates. The MCF values ranged between 16.9 μM to 134.9 μM for *P*. *brasiliensis* isolates and 33.7 μM to 67.4 μM against *P*. *lutzii* isolates (Fig 3). Thus, we reported here new experimental results showing that these molecules strongly inhibit *P*. *brasiliensis* and *P*. *lutzii* growth *in vitro*.

**Table 2:** *In vitro* antifungal activities (μg/mL) compounds interacting to the TRR1 target.

Compound	*P*. *lutzii*	Pb18	*C*. *albicans*	*C*. *parapsilosis*	*C*. *glabrata*	*C*. *tropicalis*	*C*. *neoformans*
**F0876-0030**	**32**	**32**	**128**	**>256**	**>256**	**128**	**>256**
F1109-0100	128	128	>256	>256	>256	>256	>256
**F1806-0122**	**16**	**8**	**>256**	**>256**	**>256**	**>256**	**>256**
F3010-0057	64	64	>256	>256	>256	>256	>256
F3222-4930	64	128	>256	>256	>256	>256	>256
F3307-0033	128	128	>256	>256	>256	>256	>256
**F3307-0100**	**16**	**8**	**>256**	**>256**	**>256**	**>256**	**>256**
F3394-0412	256	256	>256	>256	>256	>256	>256
F3394-0585	>256	>256	>256	>256	>256	>256	>256
F3398-5211	>256	>256	>256	>256	>256	>256	>256
F5652-2782	>256	>256	>256	>256	>256	>256	>256
F5754-0452	64	128	>256	>256	>256	>256	>256
Amphotericin B	0.250	0.250	0.5	0.5	1.0	0.5	4
Fluconazole	0.250	0.5	0.5	2.0	>4.0	2.0	ND

The biological activity of new compounds against the pathogenic fungus *Paracoccidioides* spp. has been reported for few authors, the majority describes natural compounds or derivatives. Derengowski et al. [85] showed that the MIC of farnesol (present in essential oils) for *P*. *brasiliensis* was 25 μM. Johann et al. [86] showed that compounds isolated from a Brazilian medicinal plant (*Schinus terebinthifolius*) are active against different strains of *Paracoccidiodes* spp., showing MIC values of 62.5 μg/mL (*P*. *brasiliensis*, isolate 18) and 125 μg/mL (*P*. *lutzii*, isolate Pb01). Searching for synthetic derivatives from chalcones (natural compounds) the lowest MIC value obtained was 1.9 μg/mL against isolate Pb18 [87]. When comparing the antifungal activity of F3307-0100 molecule with commercially available drugs, was observed that amphotericin B is 12 times more potent than the compounds obtained by virtual screening, on the other hand it has been associated with substantial toxicity and limitations [88], [89]. For *C*. *albicans*, *C*. *parapsilosis*, *C*. *tropicalis* and *C*. *neoformans*, no molecule showed satisfactory antifungal activity (Table 2). Differences in the full sequence may probably generate a tertiary structure with differences close to the catalytic site that may affect the binding with the compounds. The antifungal activity was observed specially for *Paracoccidioides* spp. species, and this result corroborated molecular modeling and virtual screening results, since the target used at the *in silico* analysis was from *P*. *lutzii*. As of now, the F3307-0100 compound is certainly a promising potential agent against *Paracoccidioides* spp (Table 2 and Fig 3). The antifungal activity of three compounds was dose-dependent. It will be subjected to lead optimization experiments, as the chemical structure modification could be also efficient against other human pathogenic fungi, therefore, it will hopefully contribute to the current efforts for developing more efficient antifungal drugs.

The complexes formed between the target and the three compounds with significant *in vitro* antifungal activity, F0876-0030, F1806-0122 and F3307-0100, can be visualized by Ligplot representations (Fig 2A–2C, respectively). The interaction between the compounds and TRR1 involves relevant amino acids residues at the active site such as Cys145 and/or Cys148.

### Enzymatic assays of recombinant TRR1 and TRX1 from *P*. *lutzii*

**Fig 4:**
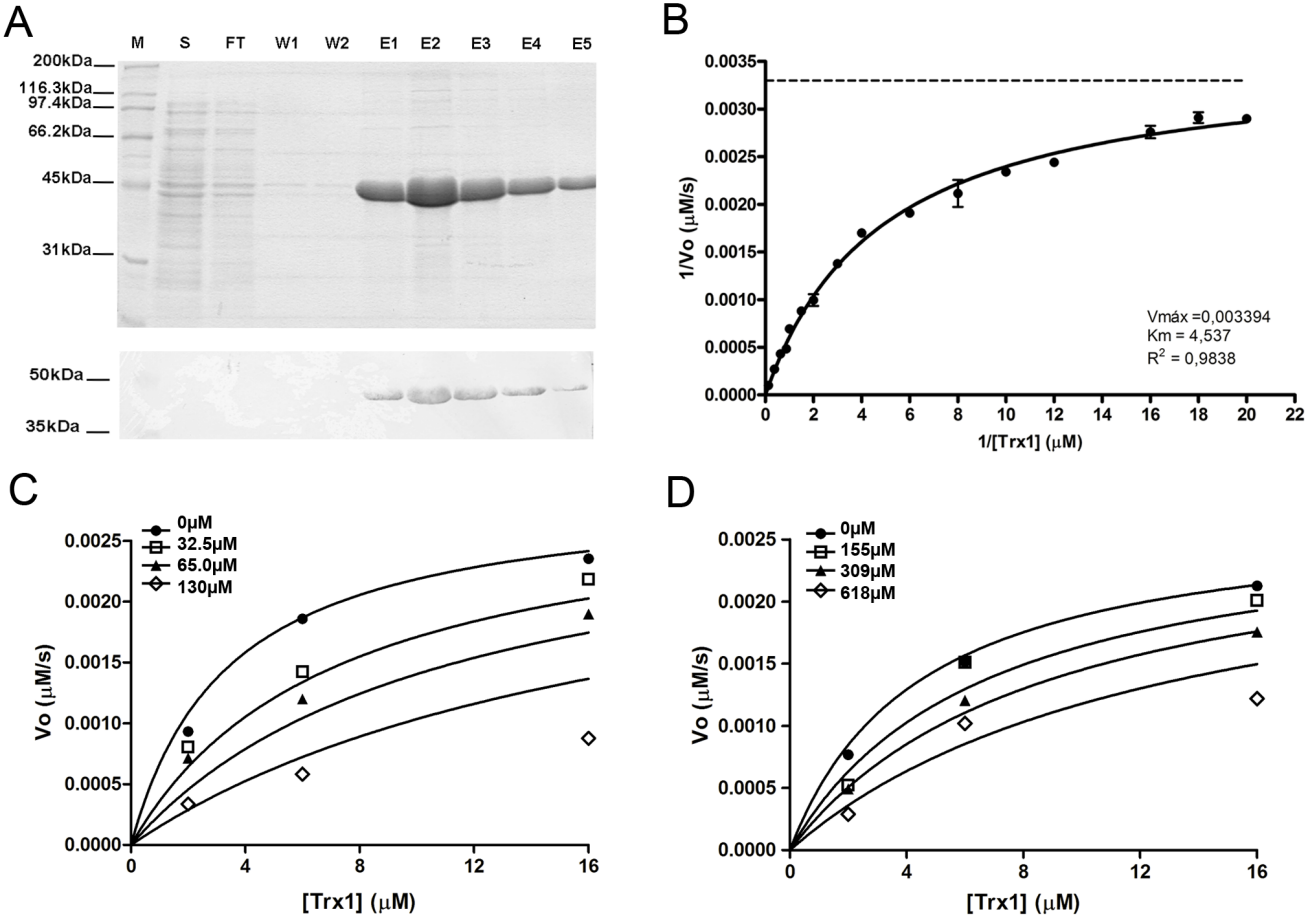
Expression, purification, enzymatic characterization and inhibition assays of recombinant TRR1 from *P*. *lutzii*. **A)** SDS-PAGE polyacrylamide gel 12% and western-blot. (S) Soluble fraction of *E*. *coli* (BL21 DE3, PET21a::*trr1*) lysate, induced with IPTG (0,5mM) for 16 hours. Flow-through (FT). Washing steps (W1 and W2). Elution steps (E1, E2, E3, E4 and E5). Molecular mass (M). **(B)** Kinetic parameters of TRR1 enzyme using recombinant Trx1 as substrate (K_m_ = 4.537μM; V_max_ = 0.003394μM/s). Inhibition assays by compounds identified by virtual screening: F0876-0030 **(C)** and F1806-0122 **(D).** The experiment was done in the presence and absence of inhibitor (μM).

In order to test if the three selected compounds were specifically inhibiting TRR1 enzyme, both TRR1 and TRX1 were purified by affinity chromatography and visualized by SDS-PAGE and western-blot (Fig 4A). The induction time of 6 hours was chosen and standardized to perform expression and downstream purification steps of recombinant TRR1 (39 kDa) and TRX1 (11 kDa), showing a high degree of purity of both enzymes.

The kinetic studies of the recombinant enzyme thioredoxin reductase (TRR1) of *P*. *lutzii* were performed using thioredoxin (Trx1), as substrate. Kinetic parameters were determined by the DTNB reduction, and the substrate concentration Trx1 was ranged from 0.125 to 20.0 μM. The kinetic parameters V_max_ and K_m_ were determined (Fig 4B), from the Michaelis-Menten equation, as 0.003394 μM/s and 4.537 μM, respectively. The reaction velocity increased gradually as the substrate concentration increased, thus showing TRR1 ability to reduce Trx1.

In order to confirm whether the selected compounds were able to specifically inhibit the recombinant TRR1 of *P*. *lutzii*, the inhibition assays were performed using Trx1 at 2.0, 6.0 and 16.0 μM concentrations. The inhibitors tested had the following concentrations: 32.5, 65 and 130 μM (F0876-0030); 155, 309 and 618 μM (F1806-0122); and 65, 130 and 260 μM (F3307-0100). Reactions were carried out in the presence and absence of inhibitor. The inhibition profile showed by Michaelis-Menten graphical representation allowed to demonstrate the TRR1 inhibition by the compounds F0876-0030 (Fig 4C) and F1806-0122 (Fig 4D). The K_m_ in the presence of varying inhibitors concentrations increased with increasing inhibitors concentrations, indicating that the inhibitors interact with the substrate active site. The experiment with the inhibitor F3307-0100 could not be considered, there was a turbidity of the solution that interfered with the absorbance reading at 412_nm_.

Inhibition assays of thioredoxin reductase enzymatic activity of the protozoan *Plasmodium falciparum*, a causative agent of tropical malaria, with specific inhibitors have shown that the more potent inhibitor has an IC_50_ value of 0,5 μM [47]. Theobald et al. [90], also working with *Plasmodium falciparum* thioredoxin reductase inhibitors, described seven specific inhibitors with IC_50_ values around 5 μM. The inhibition assays of *P*. *lutzii* TRR1 with the molecules F1806-0122 and F0876-0030 showed higher concentrations (19 μM and 260 μM, respectively), however it was possible to demonstrate that these compounds inhibit directly this drug target. Thioredoxin reductase inhibitors specific to trypanosomatids and humans diseases, as chronic lymphocytic leukemia, have been described [54], [90], [91]. However, no studies demonstrating potential inhibitors of fungi thioredoxin reductase was described. This is the first work showing specific antifungal inhibitors to TRR1 drug target.

### Cytotoxicity

**Fig 5:**
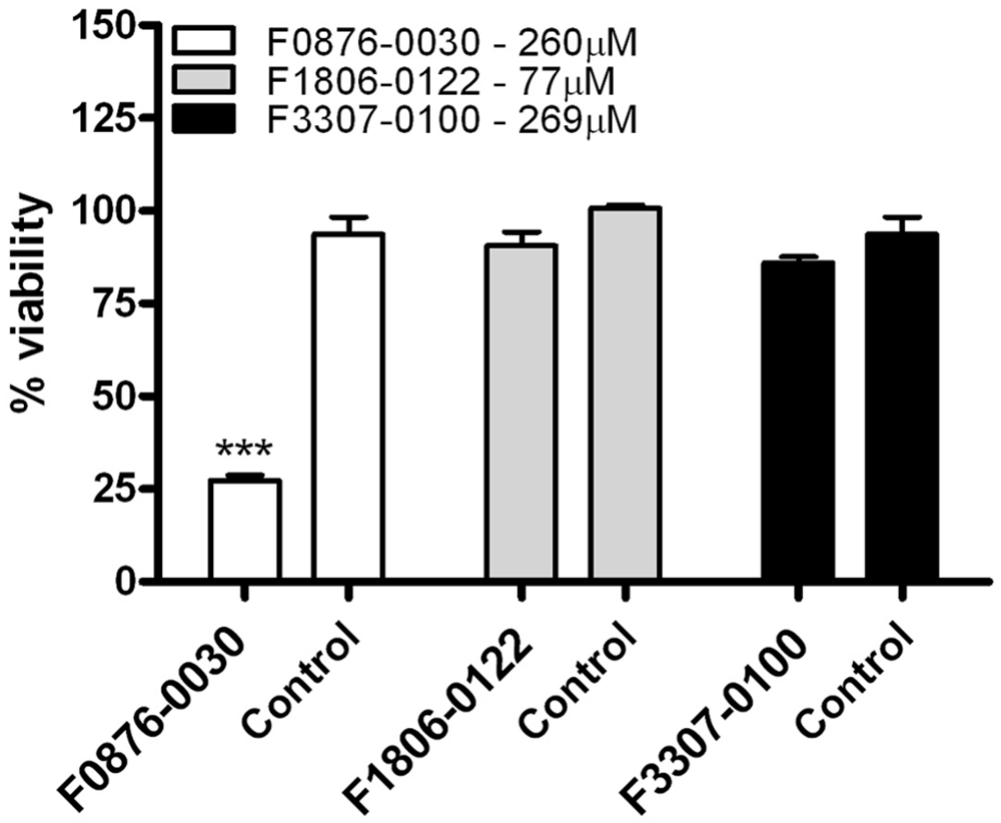
Cytotoxicity of compounds selected by virtual screening. Macrophages (J744) were incubated with the compounds were then cell viability was evaluated by a MTT assay. Results are average ± SD, n = 3.

To identify whether the small molecules were cytotoxic, the murine macrophages (J774) were exposed to the compounds with the best MIC values. Fig 5 presents the cell viability (%) as a function of compounds concentration that showed *in vitro* antifungal activities. The J774 macrophages showed more than 80% viability at concentrations lower than 260 μM (F0876-0030), 77 μM (F1806-0122) and 269 μM (F3307-0100). Two compounds (F1806-0122 and F3307-0100) showed lower cytotoxicity, and the F1806-0122 showed high antifungal activity, and lower cytotoxicity, indicating this compound to be a very promising molecule for new drug development.

## Conclusions

This work describes the identification of specific TRR1 inhibitors that providing antifungal activity against *Paracoccidioides* spp. A diverse dataset of small molecules from a commercial supplier were docked against the TRR1 model. The selected compounds from the docking predictions were tested experimentally and three compounds exhibited inhibitory activity of the fungal enzyme, demonstrating selectivity and antifungal activity against *Paracoccidioides* species. Thus, new perspectives were generated for technological development and innovation for new antifungal agents against human pathogens.
